# Relationship between adherence to the mediterranean food pattern and food self-efficacy of higher education students in Portugal: A cross-sectional study

**DOI:** 10.1371/journal.pone.0318169

**Published:** 2025-03-21

**Authors:** Bruna Brabo, António Raposo, Renata Puppin Zandonadi, Eduardo Yoshio Nakano, Zayed D. Alsharari, Thamer Alslamah, Daniela Costa, Wansoo Kim, Leandro Oliveira

**Affiliations:** 1 School of Health Sciences and Technologies, Universidade Lusófona de Humanidades e Tecnologias, Campo Grande 376, 1749-024 Lisboa, Portugal; 2 CBIOS (Research Center for Biosciences and Health Technologies), Universidade Lusófona de Humanidades e Tecnologias, Campo Grande 376, 1749-024 Lisboa, Portugal; 3 Nutrition Department, Faculty of Health Sciences, University of Brasília, Campus Universitário Darcy Ribeiro, Brasilia 70910-900, Brazil; 4 Department of Statistics, University of Brasília, Campus Universitário Darcy Ribeiro, Brasília 70910-900, Brazil; 5 Department of Clinical Nutrition, Faculty of Applied Medical Sciences, University of Tabuk, P.O. Box 741, Tabuk 71491, Saudi Arabia; 6 Department of Public Health, College of Applied Medical Sciences, Qassim University, Buraydah 51452, Saudi Arabia; 7 Institute for Medical Epidemiology, Biometrics and Informatics, Martin Luther University Halle-Wittenberg, Magdeburger Str. 8, 06112 Halle (Saale), Germany; 8 Department of Tourism Management, Gachon University, Seongnam-si, Gyeonggi-do, 13120, South Korea; 9 Polytechnic Institute of Coimbra, Coimbra Health School, Rua 5 de Outubro—S. Martinho do Bispo, Apartado 7006, 3046-854 Coimbra, Portugal; United Arab Emirates University, UNITED ARAB EMIRATES

## Abstract

When students begin their academic life, they are subject to psychological, environmental, and economic changes, which may have implications for their dietary habits. This study aims to assess the relationship between adherence to the Mediterranean food pattern (MFP), nutritional status, and food self-efficacy among a sample of higher education students in Portugal. This cross-sectional study was conducted between May and June 2023, through an online questionnaire. A total of 114 students from public and private higher education participated in this study, predominantly female (68.7%) with a median age of 23 (20; 27) years. It was found that higher body mass index (BMI), older age (p > 0.003; r: 0.273), and greater adherence to the MFP were associated with higher food self-efficacy (p > 0.003; r: 0.273). No correlations were found between the other variables. When feeling stressed, students tend to consume more sweets, fast food, and fewer fruits and vegetables. Based on the correlations between BMI, adherence to the MFP, perceived stress, and food self-efficacy, it can be concluded that higher BMI, older age, and greater adherence to the MFP are associated with higher food self-efficacy. These results can be explored for future dietary interventions in this population group.

## 1 Introduction

The transition from adolescence to adulthood, often marked by entry into higher education, is a critical period where individuals undergo significant lifestyle changes. Moving away from home, adapting to new environments, and gaining autonomy all contribute to adjustments in habits and behaviors [1,2]. This phase of life is associated with increased independence and a shift in priorities, which can lead to the adoption of new social norms and health behaviors. Unfortunately, this transition can also promote the development of unhealthy habits, influenced by factors such as the university environment, financial constraints, and academic pressures [2].

In this context, university students commonly exhibit unbalanced eating habits, such as reduced consumption of fruits and vegetables and an increased intake of foods high in fat, sugar, salt, and alcoholic beverages [3,4]. Additionally, a decline in physical activity levels [5] and irregular eating patterns due to academic and social demands further contribute to the deterioration of their dietary habits. These lifestyle changes often result in low to moderate adherence to the Mediterranean food pattern [MFP] among university students, a dietary model renowned for its health benefits [4,6,7].

The MFP is particularly significant for higher education students due to its potential to mitigate the negative health outcomes associated with poor dietary behaviors. As a model rich in bioactive compounds, it has been robustly linked to a reduced risk of chronic non-communicable diseases, including cardiovascular diseases, cancer, obesity, and type 2 diabetes mellitus [8,9]. In particular, the MFP emphasizes the consumption of plant-based foods, healthy fats, and lean proteins, which can help counteract the common nutritional deficiencies and excesses found in student diets. However, adherence to the MFP within this demographic is often hindered by lifestyle factors inherent to their academic and social environments. These include irregular schedules, financial constraints, and the social dynamics of eating (e.g., reliance on convenience foods and eating out), all of which can conflict with the principles of the MFP [2]. Such factors create barriers to adopting and maintaining this health-promoting dietary pattern, necessitating targeted interventions to address these challenges and promote sustainable dietary habits among students [3,4].

Self-efficacy, a concept introduced and developed by Albert Bandura, refers to an individual’s belief in their ability to successfully execute a specific action or task [10,11]. Those with high self-efficacy are more likely to engage in health-promoting behaviors and remain persistent in achieving their health-related goals [12]. In the field of nutrition, self-efficacy plays a pivotal role in shaping eating behaviors, influencing both overall dietary habits and adherence to specific dietary patterns, such as the Mediterranean food pattern (MFP) [13–16]. Consequently, incorporating the concept of self-efficacy into nutrition research not only deepens our understanding of dietary behaviors but also provides valuable insights for designing targeted interventions aimed at improving dietary habits and promoting healthier food choices [17].

The confidence individuals have in their ability to adhere to the MFP can be a decisive factor in sustaining commitment to the diet and, by extension, improving health outcomes. Research has shown that self-efficacy beliefs are strong predictors of healthy eating behavior, particularly with regard to adherence to structured dietary patterns like the MFP [18]. Essentially, the more confident individuals are in their capacity to follow the diet, the more likely they are to adopt and maintain healthier eating behaviors.

Supporting this idea, Warziski et al. [19] demonstrated that self-efficacy related to changing eating habits is a significant determinant of diet adherence and can contribute to positive outcomes such as weight loss. This finding emphasizes the importance of cultivating self-efficacy in promoting dietary changes, which in turn enhances health outcomes. Similarly, Anderson, Winett, and Wojcik [20] found that individuals with higher levels of self-efficacy in making healthier food choices experience improvements in nutrition quality. Those with high self-efficacy are more likely to make food choices that benefit their long-term health. Furthermore, Savoca and Miller [21] identified dietary self-efficacy as a key mediator between food preferences and eating patterns, illustrating the complex relationship between individual confidence, food choices, and dietary adherence.

Building on this background, the aim of our study is to assess the relationship between adherence to the MFP, nutritional status, and food self-efficacy among higher education students in Portugal. By examining how these factors interact, we hope to identify strategies that can effectively promote healthier dietary behaviors and improve the overall nutritional well-being of this student population.

## Materials and methods

### Procedure

This cross-sectional study was approved by the Ethics Committee of the School of Health Sciences and Technologies of the Universidade Lusófona (P11-23), on 28 April 2023. Students from public and private universities, aged 18 years or older were invited to participate. Exclusion criteria included being under 18 years old, being vegetarian/vegan, and not attending higher education in Portugal. The questionnaire took an average of 15 minutes to complete, and data collection occurred between May and June 2023. The online questionnaire was disseminated by students via institutional email, with requests to share it through their contacts and social media platforms (WhatsApp®, Facebook®, and Instagram®). The primary outreach was conducted through institutions with which the Portuguese research team had existing academic connections or networks. To minimize bias and ensure that the respondents were indeed enrolled in higher education, a mandatory initial screening question was included, requiring participants to confirm their status as higher education students. Additionally, participants were asked to provide information about their academic program and institution, which allowed for consistency checks in their responses. These measures were implemented to enhance the reliability of the data and ensure that the sample accurately reflected the intended demographic. The data were collected anonymously and saved on a secure server. Participants needed to give their written informed consent before continuing. Participating was voluntary. The participants could end the survey at any time by closing the browser tab.

### Instruments

The questionnaire was written in Portuguese and comprised four sections: socio-demographic characterization and lifestyle, dietary habits, food self-efficacy, stress, and nutrition. For this study, only the first three sections were analyzed. The sociodemographic characterization and lifestyle section included questions such as sex, age (years), nationality, area of residence (NUTSII), degree and field of study, smoking habits, alcohol consumption, caffeine intake, and anthropometric data (weight and height). Body mass index (BMI) was calculated using the formula BMI =  Weight (kg)/ Height (m)2, and the classification of nutritional status was based on the criteria of the World Health Organization [22].

The section on dietary habits included an assessment of adherence to the Mediterranean diet, specifically the Mediterranean Diet Adherence Screener (MEDAS) [23]. This tool consists of items to which a score of 0 or 1 is assigned, with a maximum score of 14 points. The higher the score, the greater the adherence to the Mediterranean dietary pattern [24]. The classification of this adherence was categorized according to the following criteria: low adherence ( ≤ 5 points), moderate adherence (6 to 9 points), and high adherence (≥10 points) [25]. For the evaluation of food self-efficacy, the Global Food Self-Efficacy Scale [GFSES], validated for the Portuguese population, was used [17]. This scale consists of 5 items with scores ranging from 0 (strongly disagree) to 4 (strongly agree), with a maximum total score of 20 points. The higher the score, the greater the food self-efficacy [17].

### Statistical analysis

The descriptive analysis included the calculation of medians and Interquartile ranges (IQR: P25; P75), as well as absolute frequencies (n) and relative frequencies (%). To assess the independence between pairs of variables, the Chi-squared test or Fisher exact test was used, while comparisons of ordered means between independent samples were conducted using the Mann-Whitney test. Additionally, Spearman correlation (r) were applied to evaluate the degree of association between pairs of continuous and ordinal variables, respectively. The null hypothesis was rejected when p <  0.05. IBM SPSS Statistics software, version 26 for Windows, was used for these analyses.

## 2 Results

A total of 134 undergraduate students from public and private universities in Portugal participated in the study. Of these, 17 were excluded because they reported being vegetarian, which invalidated the questions regarding animal product consumption in the MEDAS index, and 3 participants were excluded due to incomplete questionnaire responses. Thus, the final sample consisted of 114 students with a median age of 23 [20,26] years. The majority of the sample was female [68.4%], of Portuguese nationality (79.8%), residing in the Lisbon metropolitan area [45.6%], and pursuing a bachelor’s or integrated master’s degree (64.9%) in the health field (40.4%).

Approximately 80% of the students were non-smokers, 55.3% consumed alcoholic beverages, and 75.4% consumed coffee or coffee-containing beverages, including decaffeinated and energy drinks. Regarding nutritional status, 64.9% had a normal weight, and about 30% were overweight or obese, with only 4.4% being underweight (Table 1).

**Table 1:** Sociodemographic characterization, nutritional status, smoking habits, alcohol and caffeine consumption (n = 114).

Sociodemographic characterization			
**Age, Median (IQR)**	23 (20; 27)	**Smoking habits, n (%)**	
	**n (%)**	Non Smoking	95 (83.3)
**Sex, n (%)**		Smoker	19 (16.7)
Male	36 (31.6)	Daily consumption…, **Median (IQR)**	
Female	79 (68.4)	… of tobacco (n=14)	5.5 (3.0; 10.5)
**Nationality, n (%)**		… of electronic cigarettes (n=7)	5 (3.0; 10.0)
Portuguese	91 (79.8)		**n (%)**
Others	23 (20.2)	**Consumption of alcoholic beverages, n (%)**	
**Residence Area (NUTS II), n (%)**		No	51 (44.7)
North	13 (11.4)	Yes	63 (55.3)
Centro	31 (27.2)	Weekly consumption of…, **Median (IQR)**	
Metropolitan Area of Lisbon	52 (45.6)	… Mini beer (200ml) (n=24)	2.5 (1.0; 4.75)
Alentejo	4 (3.5)	… Bottle/can beer (330ml) (n=13)	4 (2.0; 7;5)
Algarve	13 (11.4)	… Beer glass (250ml) (n=23)	3 (1.0: 4.0)
Autonomous Region of the Azores	1 (0.9)	… Wine (150ml) (n=26)	2 (1.0; 3.25)
**Educational Cycle Attended, n (%)**		… Bottle/can cider (330ml) (n=19)	1 (1.0; 2.0)
Postgraduate and Specialization	7 (6.1)	… Distilled drinks (50mL) (vodka, rum, whiskey, liqueur, cognac, gin, spirits…) (n=20)	1 (1.0; 2.0)
Licenciature	74 (64.9)		
Master	25 (21.9)		
Doctorate	8 (7.0)	**Consumption of caffeinated drinks, n (%)**	
**Course Area, n (%)**		No	28 (24.6)
Health	46 (40.4)	Yes	86 (75.4)
Life sciences	18 (15.8)	Weekly consumption of…, **Median (IQR)**	
Art	7 (6.1)	…Decaffeinated (n=18)	2 (1.0; 3.5)
Social and behavioral sciences	10 (8.8)	… Cafes (n=75)	7 (3.0; 14;0)
Other	33 (28.9)	… Hot drinks with caffeine (excluding coffee): gallon, cappuccino, mocha, etc… (n=44)	3 (2.0;7.0)
**Nutritional Status, n (%)**		… Energy drinks with caffeine (Red Bull®, Monster®.) (n=10)	1.5 (1.0; 4.0)
Low weight	5 (4.4)		
Normal weight	74 (64.9)		
Overweight	29 (25.4)		
Obesity	6 (5.3)		

Table 2 presents the data regarding adherence to the MFP. It was observed that olive oil is the main source of culinary fat used by students [93%]; however, 78.1% consume less than four tablespoons of olive oil per day. About 60% of participants consume fewer than 2 servings of vegetables daily and fewer than 3 pieces of fruit (including natural fruit juices) daily. Most students (85.1%) consume more than 1 serving of red meat, hamburgers, or meat products (ham, sausages, etc.) daily, while 55.3% consume more than 1 serving of butter, margarine, or cream daily.

**Table 2:** Adherence to the Mediterranean food pattern (n = 114).

	Criteria for 1 point		1 point		
**n (%)**		**Total (n = 114)**	**Female (n = 78)**	**Male** **(n = 36)**	***p***
1. Do you use olive oil as your main culinary fat?	Yes	106 (93.0%)	72 (92.3%)	34 (94.4%)	1.000^b^
2. How much olive oil do you consume in ^a^ day (including for frying, seasoning, salad dressing, meals eaten outside the home, etc.)?	≥ 4 Tablespoons	25 (21.9%)	15 (19.2%)	10 (27.8%)	0.305^a^
3. How many servings of vegetables do you consume per day?	≥2	46 (40.4%)	35 (44.9%)	11 (30.6%)	0.149^a^
4. How many pieces of fruit (including natural fruit juices) do you consume per day?	≥3	43 (37.7%)	33 (42.3%)	10 (27.8%)	0.137^a^
5. How many servings of red meat, hamburgers, or meat products (ham, sausage, etc.) do you consume per day?	<1	17 (14.9%)	15 (19.2%)	2 (5.6%)	0.057^a^
6. How many servings of butter, margarine, or cream do you consume per day?	<1	51 (44.7%)	34 (43.6%)	17 (47.2%)	0.717^a^
7. How many sugary or carbonated beverages do you drink per day?	<1	88 (77.2%)	65 (83.3%)	23 (63.9%)	0.021^a^^*^
8. How many glasses of wine do you drink per week?	≥7 cups	2 (1.8%)	1 (1.3%)	1 (2.8%)	0.534^b^
9. How many servings of pulses do you consume per week?	≥3	64 (56.1%)	45 (57.7%)	19 (52.8%)	0.623^a^
10. How many servings of fish or seafood do you consume per week?	≥3	52 (45.6%)	36 (46.2%)	16 (44.4%)	0.865^a^
11. How many times per week do you consume commercial bakery products or sweets (non-homemade), such as cakes, cookies, biscuits?	<3	67 (58.8%)	45 (57.7%)	22 (61.1%)	0.730^a^
12. How many servings of nuts (walnuts, almonds, including peanuts) do you consume per week?	≥3	30 (26.3%)	19 (24.4%)	11 (30.6%)	0.485^a^
13. Do you preferentially consume chicken, turkey, or rabbit instead of beef, pork, hamburger, or sausage?	Yes	80 (70.2%)	60 (76.9%)	20 (55.6%)	0.020^a^^*^
14. How many times per week do you consume vegetables, pasta, rice, or other dishes cooked with ^a^ sautéed base (tomato, onion, leek or garlic, and olive oil)?	≥2	105 (92.1%)	72 (92.3%)	33 (91.7%)	1.000^b^
Adherence to the Mediterranean food pattern^**^ **n (%)**		**Total** **(n = 114)**	**Female** **(n = 78)**	**Male** **(n = 36)**	***p***
	Low adherence	15 (13.2%)	7 (9.0%)	8 (22.2%)	
	Moderate adherence	89 (78.1%)	62 (79.5%)	27 (75.0%)	0.064^c^
	High adherence	10 (8.8%)	9 (11.5%)	1 (2.8%)	

* *p* < 0.005.

^a^Chi-squared test;

^b^Fisher’s exact test;

^c^Chi-squared test via Monte Carlo simulation;

** Low adherence: up to 4 points; Moderate Adherence: 5 to 9 points; High adherence: 10 or more points.

Regarding beverages, 22.8% of students consume sugary or carbonated drinks daily, and 98.2% consume less than 7 glasses of wine per week. Over 50.0% of participants consume 3 or more servings of legumes per week, while 54.4% do not consume 3 or more servings of fish or seafood per week. Regarding commercial bakery products or sweets (non-homemade), 60.0% of students consume this type of food less than 3 times per week; on the other hand, only 26.3% consume 3 or more servings of nuts (walnuts, almonds, including peanuts) per week. Approximately 70% of students prefer chicken, turkey, or rabbit over beef, pork, hamburgers, or sausages. Finally, more than 90% consume 2 or more times per week vegetables, pasta, rice, or other dishes cooked with a sautéed base (tomato, onion, leek or garlic, and olive oil). Most participants (78.1%) demonstrate moderate adherence to the MFP. It was also found that females consume more sugary or carbonated beverages daily than males (p = 0.021). Additionally, females are more likely to prefer chicken, turkey, or rabbit over beef, pork, hamburger, or sausage than males (p = 0.020).

Global food self-efficacy presented a significant correlation with adherence to the Mediterranean food pattern (Table 3). More than half of the participants strongly agree/agree very much with the statements: “I give up controlling my diet when I encounter difficulties,” “I face and solve problems related to controlling my diet,” and “I am persistent in solving difficulties in controlling my diet.” On the other hand, more than half of the students do not agree or slightly agree with the statement “I am quick to make decisions and implement measures to control my diet.” Additionally, approximately 40% of students strongly agree/agree very much with the statement “I always find energy to over-come difficulties in controlling my diet.” The median (P25; P75) of the GFSES was 15 (14; 16).

**Table 3:** Global Food Self-Efficacy Scale and its association with adherence to Mediterranean food pattern (n = 114).

	Not Agree n (%)	Agree Little n (%)	Agree Moderately n (%)	Agree Very Much n (%)	Agree Extremely n (%)	Correlation^*^ (p)
“I give up controlling my diet when I encounter difficulties.”	2 (1.8%)	13 (11.4%)	43 (37.7%)	33 (28.9%)	23 (20.2%)	0.332 (<0.001)
“I am quick to make decisions and implement measures to control my diet.”	31 (27.2%)	37 (32.5%)	23 (20.2%)	16 (14.0%)	7 (6.1%)	-0.302 (0.001)
“I face and resolve problems related to controlling my diet.”	4 (3.5%)	17 (14.9%)	36 (31.6%)	35 (30.7%)	22 (19.3%)	0.399 (<0.001)
“I am persistent in resolving difficulties in controlling my diet.”	2 (1.8%)	26 (22.8%)	31 (27.2%)	31 (27.2%)	24 (21.1%)	0.409 (<0.001)
“I always find energy to overcome difficulties in controlling my diet.”	4 (3.5%)	28 (24.6%)	40 (35.1%)	21 (18.4%)	21 (18.4%)	0.319 (0.001)
Global Food Self-Efficacy Scale	**Median**	**IQR** ^**^				
	15	(14; 16)				0.251 (0.007)

* Spearman correlation with Adherence to the Mediterranean Food Pattern (whole scale).

** IQR: Interquartile Range.

The correlation analysis revealed a positive correlation between BMI and age (p =  0.008; r =  0.246), as well as between adherence to the Mediterranean Food Pattern (MFP) and food self-efficacy (p =  0.002; r =  0.286). Additionally, a negative correlation was observed between BMI and food self-efficacy (p =  0.024; r =  -0.212) – Table 4.

**Table 4:** Correlation between age, body mass index, mediterranean diet adherence screener, and global food self-efficacy scale (n = 114).

		Age (years)	Body Mass Index	Mediterranean Diet Adherence Screener	Global Food Self-Efficacy Scale
**Age (years)**	r	1.000	0.246^*^	0.121	0.113
	*p*	---	0.008	0.198	0.233
	n	---	114	114	114
**Body Mass Index**	r	---	1.000	-0.002	-0.212^*^
	*p*	---	---	0.984	0.024
	n	---	---	114	114
**Mediterranean Diet Adherence Screener**	r	---	---	1.000	0.286^*^
	*p*	---	---	---	0.002
	n	---	---	---	114
**Global Food Self-Efficacy Scale**	r	---	---	---	1.000
	*p*	---	---	---	---
	n	---	---	---	---

* *p* < 0.005; *r*: Spearman correlation.

## 3 Discussion

This cross-sectional study explored the connections between adherence to the MFP, food self-efficacy, and the nutritional status of undergraduate students in Portugal. The primary aim was to evaluate how adherence to this dietary pattern, renowned for its numerous health benefits, aligns with students’ confidence in making healthy dietary choices and how these factors influence their overall nutritional status.

The findings indicated that most participants had a normal BMI, which could be attributed to their adherence to the MFP. This contrasts with studies demonstrating that low adherence often correlates with higher BMI percentages [27]. Differences may stem from varying dietary habits among university students. Numerous investigations, including this one, have highlighted an inverse relationship between adherence to the MFP and BMI [26,28], and food self-efficacy [21]. According to our data, most students exhibited moderate adherence to the Mediterranean diet, with low adherence being the second most common trend. This is consistent with other studies [29] and national data revealing that only 26% of the Portuguese population demonstrates high adherence to the MFP [30]. When compared to other Portuguese studies, the prevalence of high adherence in this research was lower. For example, a study involving 759 students from the University of Porto found a 21.3% high adherence rate [31], while another involving 305 students from Lusófona University indicated a 12.5% prevalence [6]. Similarly, Graça et al. [32] reported 8.2% high adherence among higher education students and researchers. Nationally, broader studies including 480 students and adults observed an 11% prevalence of high adherence.

International comparisons provide additional insights. For instance, a study involving 584 Spanish university students reported a 36.4% high adherence to the MFP, with notable gender differences in beverage consumption patterns [4]. Contrastingly, our study observed low wine consumption, which negatively impacted adherence scores. While this might initially appear beneficial given Portugal’s high alcohol consumption rates [33], it raises concerns as other alcoholic beverages might substitute wine. In Peru, university students demonstrated a high adherence rate of 14.2% [26], whereas a Lebanese study reported a 41.0% rate but highlighted low intakes of vegetables, fish, and nuts, coupled with a preference for white meats and refined products, paralleling our findings [34].

These patterns may be influenced by the gradual replacement of traditional dietary habits with a Westernized diet prevalent in Europe [7]. Additionally, individual dietary choices shaped by trends like weight-loss diets, meal skipping, or frequent consumption of snacks, soft drinks, and processed foods play a significant role [35]. Oliveras López and Nieto Guindo [36] emphasize that the Western diet is characterized by a low intake of nutrient-dense foods like fruits and vegetables and a high intake of processed items rich in sugars, animal fats, and red meat [37]. Structured dietary assessments are essential for deriving meaningful conclusions [38].

No studies have specifically investigated why females consume more sugary or carbonated drinks than males. Contradicting our findings, other research suggests that males tend to consume these beverages more frequently [39–42]. Understanding these gender-based differences in beverage consumption is crucial for nutrition professionals and policymakers [40,41].

Gender-based dietary preferences were also observed, with females favoring lean meats like chicken, turkey, or rabbit, while males preferred beef, pork, and processed meats such as sausages and hamburgers. These preferences contribute to the higher prevalence of overweight and obesity among males, as highlighted in previous research [38]. Emotional eating, driven by stress or anxiety, is another factor influencing unhealthy food choices, often leading to increased consumption of sugary and processed items [43]. This lack of control over food intake may stem from inadequate emotional coping mechanisms among students [44].

Our study also underscored the impact of stress on dietary habits, with students consuming fewer fruits and vegetables during stressful periods. This observation aligns with other studies documenting a decline in fruit and vegetable intake and a rise in fast-food consumption among university students [45–47]. Dietary patterns established during university years can persist, carrying long-term health implications [48].

In Brazil, a study examining stress among young adults identified psychological symptoms, such as heightened emotional sensitivity, as primary responses. Females were more likely to report feelings of worry, lack of control, and being overwhelmed by challenges [49]. Greater adherence to the Mediterranean diet has been associated with enhanced food self-efficacy, as individuals struggling with emotional regulation often resort to comfort eating and deviate from healthy dietary practices [50]. Universities play a pivotal role in promoting healthy eating habits by providing balanced and varied menus in campus dining facilities, as supported by studies [51–54].

## Limitations and future directions

While this study provides valuable insights into the dietary habits of higher education students in Portugal, several limitations must be critically considered, as they influence the interpretation and generalizability of the findings. Firstly, the cross-sectional design employed in this study limits our ability to establish causal relationships between the observed variables. By capturing data at a single point in time, the study fails to account for potential changes in dietary behaviors over time or the influence of external factors that may impact students’ eating habits. This temporal limitation makes it difficult to infer whether the relationships identified in the study are a cause or merely an association, highlighting the need for longitudinal research to explore these dynamics further.

Additionally, the convenience sampling method used in this study, alongside the relatively small sample size, raises concerns about the representativeness of the findings. The sample, which is predominantly concentrated in Lisbon, may not accurately reflect the broader population of higher education students across Portugal. As such, the generalizability of the results to students in other regions or universities is limited. The use of convenience sampling also introduces potential selection bias, as it may not capture the full diversity of student demographics, potentially skewing the results. Future studies should aim to employ more rigorous and randomized sampling techniques to ensure a more representative and diverse sample, improving the external validity of the findings. However, it is worth noting that similar sample sizes are common in pilot studies, as referenced by previous literature [55–57].

Another limitation of the study is the exclusion of vegetarians from the analysis due to challenges in adapting the MEDAS to accurately reflect their dietary patterns, particularly in relation to meat consumption. This exclusion reduces the applicability of the MEDAS to vegetarian students, whose dietary habits may differ significantly from those of non-vegetarians. As a result, the dietary patterns observed in this study may not fully capture the diversity of eating habits among all higher education students, particularly those following plant-based diets. Future research should consider developing or adapting dietary adherence tools to include vegetarians and other dietary subgroups, ensuring that the full spectrum of student eating behaviors is represented.

Despite these constraints, the study has notable strengths. The use of validated questionnaire items ensures the reliability and accuracy of the data, particularly in assessing dietary habits and food self-efficacy. Furthermore, leveraging social media for data collection enabled the inclusion of participants from diverse academic levels, courses, and geographic regions, enhancing the heterogeneity of the sample. Importantly, the study identified significant associations between BMI, adherence to the MFP, and food self-efficacy, offering valuable insights into the interplay between dietary patterns and health-related behaviors among young adults in higher education.

Future research should address these limitations by adopting longitudinal designs to better explore causal relationships and the evolution of dietary habits over time. Expanding the sample to include larger and more geographically diverse populations, as well as subgroups such as vegetarians, will enhance the representativeness and inclusivity of findings. Additionally, future studies could investigate the role of unmeasured factors, such as socioeconomic status and psychological determinants, to provide a more comprehensive understanding of dietary behaviors in this population. These efforts will further strengthen the evidence base for developing targeted interventions aimed at improving dietary habits and health outcomes among higher education students.

## 4 Conclusions

Approximately 30% of the students in our study are classified as overweight or obese, and only 8.8% exhibit high adherence to the MFP. The median score obtained on the Global Food Self-Efficacy Scale was 15 (14; 16) points, indicating varying levels of confidence in making healthy food choices. Our analysis revealed several important associations. Firstly, a higher BMI is linked to older age among the student population. This suggests that weight management may become increasingly challenging as students age, potentially due to lifestyle changes or increased academic pressures. Secondly, greater adherence to the MFP is positively correlated with higher food self-efficacy. Students with greater confidence in their ability to make healthy dietary choices are more likely to adhere closely to the MFP, which has been associated in research with potential benefits, such as a reduced risk of certain chronic diseases. These results underscore the potential benefits of promoting the MFP among higher education students. Specifically, interventions tailored to younger undergraduates, who may benefit from early engagement, could be particularly impactful. Educational campaigns and cooking workshops could directly address low self-efficacy by equipping students with practical skills and knowledge to make healthier food choices. Additionally, providing accessible and affordable Mediterranean diet options on campus may help bridge the gap between dietary awareness and practice. Evidence from similar successful interventions at other institutions supports the feasibility and effectiveness of such approaches, further justifying their implementation.

## Supporting information

S1 Data minimal data set.
(XLSX)

S1 File PLOS’ questionnaire on inclusivity in global research.
(DOCX)
